# Recent Advances in the Synthesis of Xanthines: A Short Review

**DOI:** 10.1155/2022/8239931

**Published:** 2022-11-08

**Authors:** Anandi Kapri, Nitin Gupta, Sumitra Nain

**Affiliations:** ^1^Department of Pharmacy, Banasthali Vidyapith, Banasthali, Rajasthan, India; ^2^Agilent Technologies Pvt. Ltd., 181/46, Industrial Area, Phase-1, Chandigarh, India

## Abstract

Xanthine and its derivatives are considered a pharmacologically potential moiety that manifests immense biological activities. Owing to this much diversity in the biological field, this scaffold has fascinated the attention of many researchers around the globe to scrutinize its basic structure chemically as well as biologically. In recent years, xanthine derivatives have been used therapeutically in different pathological conditions due to their presence in day-to-day life. Herein, we review the recent progress in the synthesis of xanthine and its derivatives. Some of the widely used synthetic strategies such as (a) Traube's synthesis, (b) one-pot synthesis, (c) xanthine-anneleated synthesis, and (d) miscellaneous synthesis were compiled in this review paper. The results obtained from this review paper highlight the significance of various xanthine derivatives as possible leads to the development of new drugs. The data compiled in this review paper could help the medicinal chemist in designing new active compounds from the modification of the already existing compounds in the search for novel drug leads. This report concludes that the various synthetic procedures exemplified in this review paper may serve as a support system for the designing of new molecules with a xanthine scaffold. Thus, we hope that this molecule may serve as the prototype in order to find out more active xanthine derivatives.

## 1. Introduction

**Figure 1:**
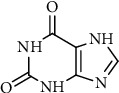
Structure of xanthine.

Xanthine or 3,7-dihydropurine-2,6-dione (see Figure 1), a unique heterocycle is a purine base containing nitrogen as a central atom and composed of a pyrimidine ring fused with an imidazole ring.

It is an essential core element of diverse natural products because their structural fragments are found in various natural and synthetic medicinally active compounds [1]. The versatility of the xanthine moiety displays that it is the essential part of several medicinal agents and some of its derivatives have shown innumerable physiological and pharmacological activities viz. respiratory tract, heart, smooth muscle cells, CNS (central nervous system), kidney, and stomach [2]. In brief, the objective of such studies is to expose the drug-like properties of xanthine and its derivatives in order to build prospects for harnessing the full potential of this scaffold.

Xanthine scaffold has fascinated the attention of researchers in health sciences due to its remarkable properties either chemical or physical [3]. Over the last two decades, the compound and its derivatives have gained considerable interest [4]. The xanthine scaffold can also act as a basic framework for numerous pharmacologically active scaffolds [5]. Several patent applications were also filed for xanthine derivatives as mentioned in (see Table 1)**, w**hich displays the therapeutic effectiveness of this scaffold.

Owing to the importance of xanthine moiety in medicinal chemistry and its broad range of biological activity [22]. This review article primarily focuses on the updated knowledge of synthetic methods used to access xanthine scaffolds. In the present work, we have compiled the recent literature that belongs to the synthetic strategies of these derivatives.

## 2. Search Strategy

The data has been compiled from the year 2010 to 13 July 2022. We performed an electronic search to find out the existing literature on xanthine derivatives. For this purpose, the compiled data has been searched from different search engines and databases such as Science Direct, Google Scholar, Cochrane, PubMed, Scopus, and Scientific information databases. More than ten months were completely used to compile data for this manuscript. The key terms used during the search were “xanthine,” “xanthine derivatives” “synthesis of xanthines” and “targets of xanthine derivatives.” One hundred twenty-two papers were screened and then inclusion and exclusion criteria were applied to prepare this manuscript. Out of these papers, forty-four papers were used to prepare this manuscript. The last search was conducted on 15. March 2022.

The results obtained from this review paper highlight the significance of various xanthine derivatives as possible leads to the development of new drugs. The reactions covered in this review fall under the following categories: (a) Traube's synthesis, (b) one-pot synthesis, (c) xanthine-anneleated synthesis, and (d) miscellaneous synthesis.

## 3. Synthesis Methods

### 3.1. Traube's Method

**Scheme 1:**
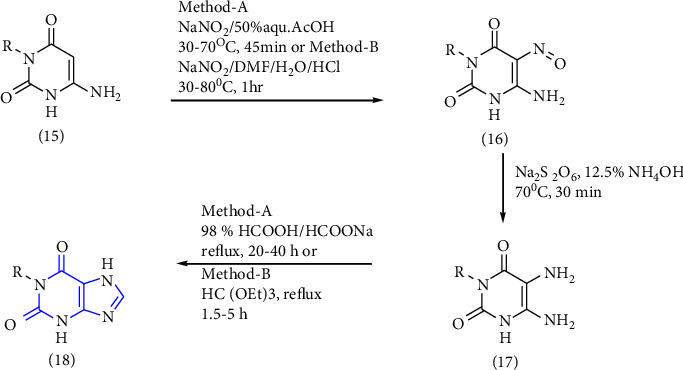
Synthetic procedure for substituted xanthines by Traube's method.

Traube's synthesis is the oldest and most used synthesis method for xanthine analogs. The method consists of the preparation of 5,6-diaminouracil **(17)** from urea or substituted urea. Several xanthine derivatives were synthesized by using uracil **(15)** as a precursor. Two methods were employed for the synthesis. According to method A-the nitrosation reaction **(16)** took place in presence of sodium nitrite and acetic acid followed by reduction **(17)** in presence of sodium dithionate and cyclisation to form substituted xanthine in presence of formic acid and sodium formate. In method B, the nitrosation reaction took place in presence of sodium nitrite, dimethyl formate, and hydrochloric acid followed by reduction and ring closure in presence of triethylorthoformate to form substituted xanthine **(18)**. The main disadvantage of this method is that the overall process is tedious because of the ring closure synthetic mechanism (Scheme 1). Some recently used examples of this synthesis method are discussed in this section.

**Scheme 2:**
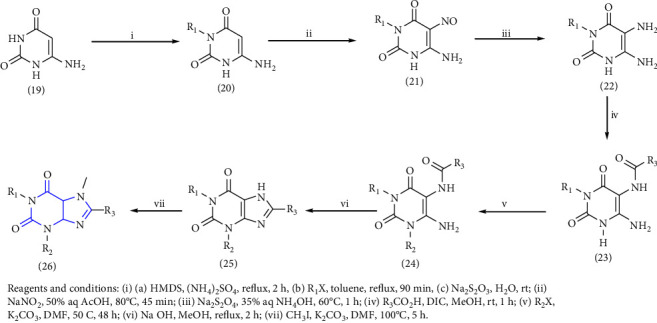
Synthesis of 1-,3-,8-trisubstituted 1H-purine-2,6(3H,9H)-diones.

The preparation of 1-,3-,7-,8-substituted xanthines was done by using 3-substituted-6-aminouracil **(19)** as the substrate. This substrate was further alkylated **(20)** with alkyl iodide, followed by nitrosation **(21)** with sodium nitrite and reduction lead to 3-substituted 5,6-diaminouracil **(22-24)**, followed by subsequent condensation with corresponding carboxylic acid lead to the corresponding xanthines **(25, 26)** [23] (Scheme 2). The derivatives were evaluated for the A1, A2A, A2B, and A3 subtypes of adenosine receptors. A number of derivatives have shown moderate to high affinity for human A1 and A2B adenosine receptors and lower affinity for A2A and A3 adenosine receptors.

**Scheme 3:**
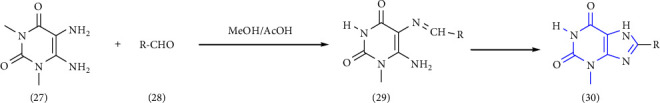
Synthesis of 8-cyclopentyloxyphenyl xanthine derivatives.

An efficient method was reported for the synthesis of 8-substituted xanthines. The synthesis was performed by using 5, 6-diamino-1, 3-dimethyluracil **(27)** as starting material. The starting material was synthesized by condensing N, N-dimethyl urea, cyanoacetic acid, and acetic anhydride, and subsequent nitrosation and reduction afforded 5, 6-diamino-1, 3-dimethyluracil. Treatment of this compound with a substituted aldehyde **(28)** forms benzylidene derivative **(29)**, which on oxidative cyclisation, by refluxing with thionyl chloride afforded the desired xanthine derivative **(30)** [24] (Scheme 3).

**Scheme 4:**
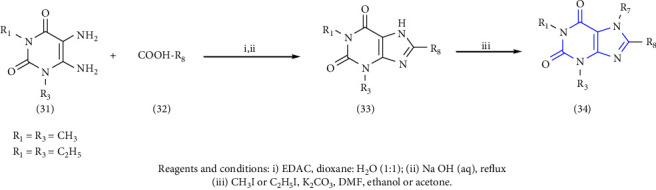
Synthetic pathway to xanthine derivatives.

A series of 8-substituted xanthines by using 1,3-dimethyl and 1, 3-diethyl 5, 6-diaminouracils **(31)** as starting compounds was reported. The uracil was treated with desired carboxylic acid **(32)** to yield intermediate amides. Furthermore, the amide was reacted with NaOH (sodium hydroxide) to yield 1-, 3-, 7-trisubstituted xanthines **(33)**. The obtained compound was further treated with an excess of iodomethane or iodoethane to yield 1-, 3-, 7-, 8-substituted xanthines **(34)** [25] ( Scheme 4).

**Scheme 5:**
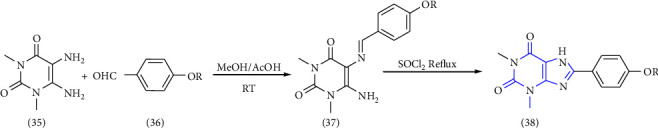
Synthesis of 8-(p-substituted) xanthine derivatives.

Novel 8-(p-substituted) xanthine derivatives were designed, synthesized, and reported. The synthesis was done by using 5, 6-diamino-1, 3-dimethyluracil **(35)**. The treatment of uracil with aldehydes **(36)** yields the corresponding Schiff base **(37)**, and further ring closure afforded the desired xanthine derivatives **(38)** [26] (Scheme 5).

**Scheme 6:**
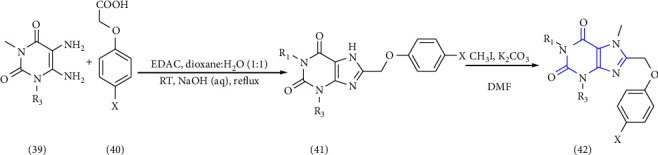
Synthesis of 8-(phenoxymethyl)-xanthine analogs.

The synthesis of 1, 3, 7, 8-substituted xanthine derivatives was reported. The synthesis was done by reacting 1,3-dialkyl-5, 6-diaminouracil **(39)** with that of substituted phenoxy acetic acid **(40)** and EDAC (1-ethyl-3-(3-dimethylaminopropyl)-carbodiimide). In the next step, the obtained intermediate amide was treated with aq. NaOH to yield the desired xanthine analog **(41, 42)** [27] (Scheme 6).

**Scheme 7:**
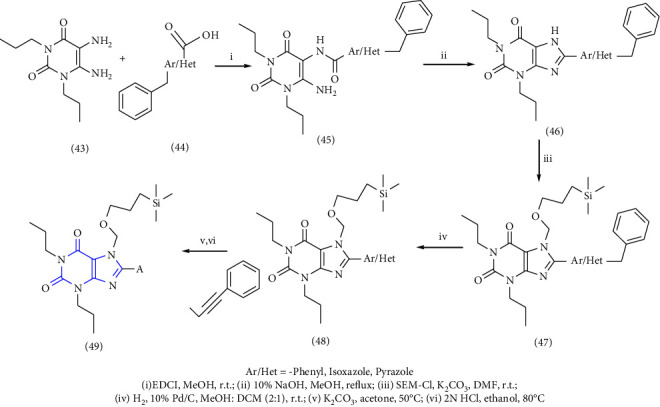
Synthetic scheme of 1-,3-,8-substituted xanthines.

1-, 3-, 8-substituted xanthines were synthesized from 1,3-dipropyl-5, 6-diaminouracil **(43)**. The uracil was reacted with corresponding carboxylic acid **(44)** in presence of EDCI [1-ethyl-3-(3-dimethylaminopropyl) carbodiimide] to yield the benzylidene derivative **(45)**, and further ring closure was achieved with NaOH to give 8-substituted xanthines **(46-49)** [28] (Scheme 7).

**Scheme 8:**
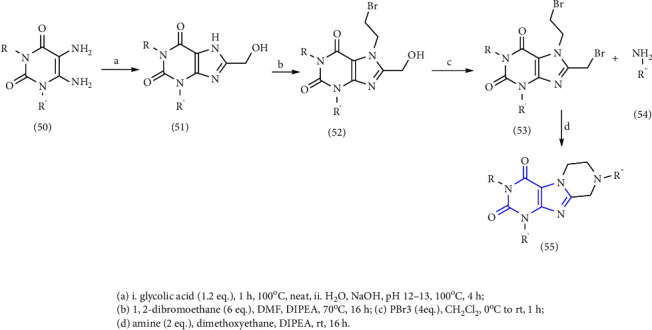
Schematic pathway of 8-substituted purinediones.

A new reaction for the synthesis of tetrahydropyrazino [2,1f] purinediones was reported. The 5,6-diaminouracil **(50)** was treated with glycolic acid at 100°C, ring closure was achieved with NaOH solution to give 8-hydroxymethylxanthine **(51)**. Subsequent alkylation **(52)** at position-7 in the presence of DIPEA (N, N-diisopropylethylamine). In the end, the obtained compound **(53)** was treated with various amines **(54)** resulting in xanthine derivatives **(55)** [29] (Scheme 8).

**Scheme 9:**
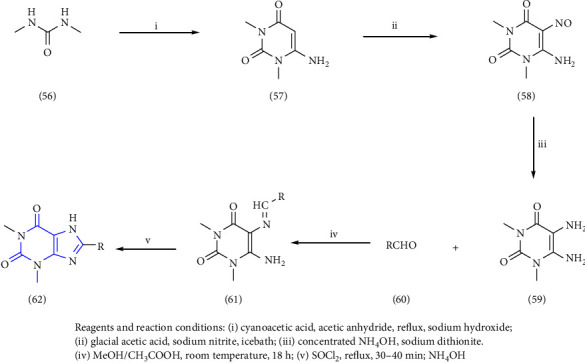
Synthesis of 8-substituted xanthines.

8-(2-nitroaryl) xanthines were synthesized from dimethyl urea **(56)**, cyanoacetic acid, and acetic anhydride. The nitrosation (**57)** and reduction of 6-aminouracil **(58)** was achieved resulting in 5, 6-diaminouracil **(59)**, which on further treatment with a corresponding aldehyde **(60)** yielded benzylidene derivative **(61)**. These derivatives were further cyclized by refluxing in thionyl chloride to give the required xanthines **(62)**. All the synthesized compounds were further biologically evaluated for adenosine receptor subtypes [30] (Scheme 9).

**Scheme 10:**
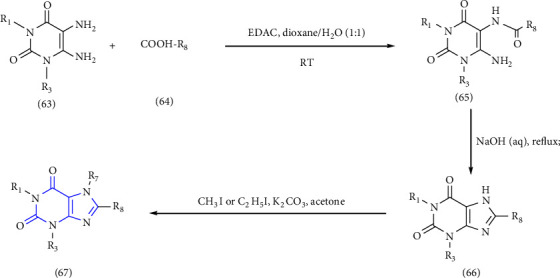
Synthesis of 8-(p-substituted) xanthines.

A novel series of 1-,3-,7-triethyl substituted xanthine derivatives were reported. The 5,6-diamino-1,3-dialkyluracil **(63)** was used as the starting material. The starting material was treated with commercially available carboxylic acid **(64)** to give 1,3-dialkyl-6-amino-5-carboxamidouracil **(65)** intermediate, subsequent cyclisation of this derivative yield corresponding xanthine derivatives **(66,67)** [31] (Scheme 10).

**Scheme 11:**
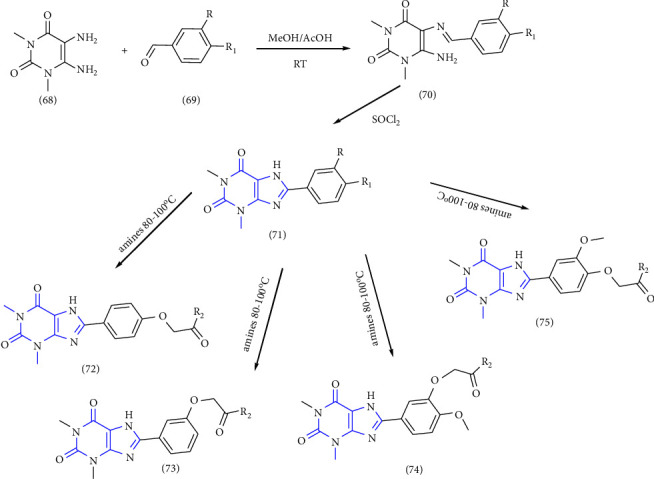
Systematic study for the synthesis of xanthine carboxylate amides.

8-phenyl-1,3-dimethylxanthine derivatives were synthesized and reported. In this synthesis 5,6 diaminouracil **(68)** was used as the key intermediate. The intermediate was treated with a different substituted aldehyde **(69)** and methanol: acetic acid (4 : 1) results in the formation of benzylidene derivative **(70)**, which further gets cyclized to form xanthine carboxylate ester **(71)**, which on treatment with substituted amines at 80–100°C to yield **(72-75)** [32] (Scheme 11).

**Scheme 12:**
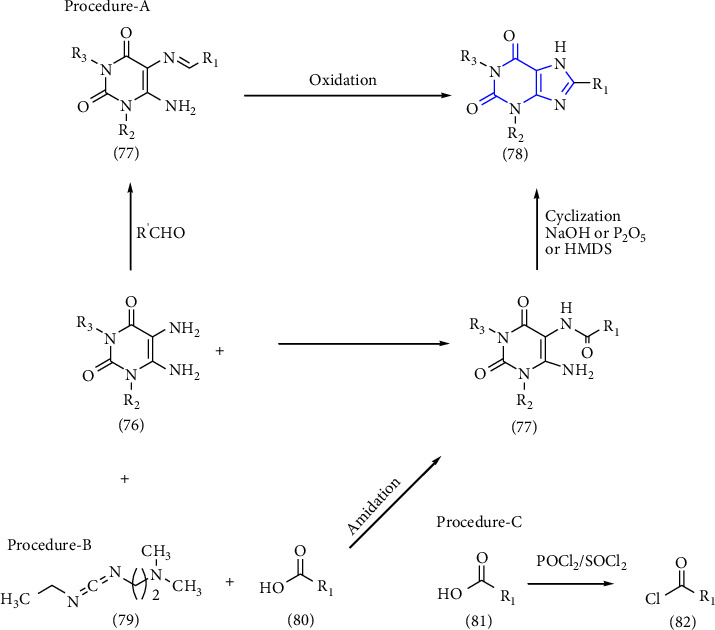
Synthesis of 8-substituted xanthine derivatives.

The fast, efficient synthesis of 6-Amino-5-carboxamidouracils as the predecessor for the 8-substituted xanthines was reported. In procedure A: the 5,6-diaminouracils **(76)** were condensed with aldehydes, results in imine formation **(77)**, following oxidative cyclisation resulted in 8-substituted xanthine derivatives **(78)**, which is the most common route of xanthine synthesis. In Procedure B, the uracil **(76)** derivative was treated with carboxylic acid **(80)** and EDAC-HCl (1-ethyl-3-(3-dimethylaminopropyl)) carbodiimide hydrochloride **(79)** which lead to the formation of xanthine derivative **(78)**. In Procedure-C, the 5,6-diaminouracils **(76)** were made to react with carboxylic acid **(81)** but prior to this reaction the activation of the carboxylic acid to form carboxylic acid chloride **(82)** was done, further leading to the formation of 6-amino-5-carboxamidouracil derivative **(77)** and then subsequent ring closure yield 8-substituted xanthines **(73)** [33] (Scheme 12).

**Scheme 13:**
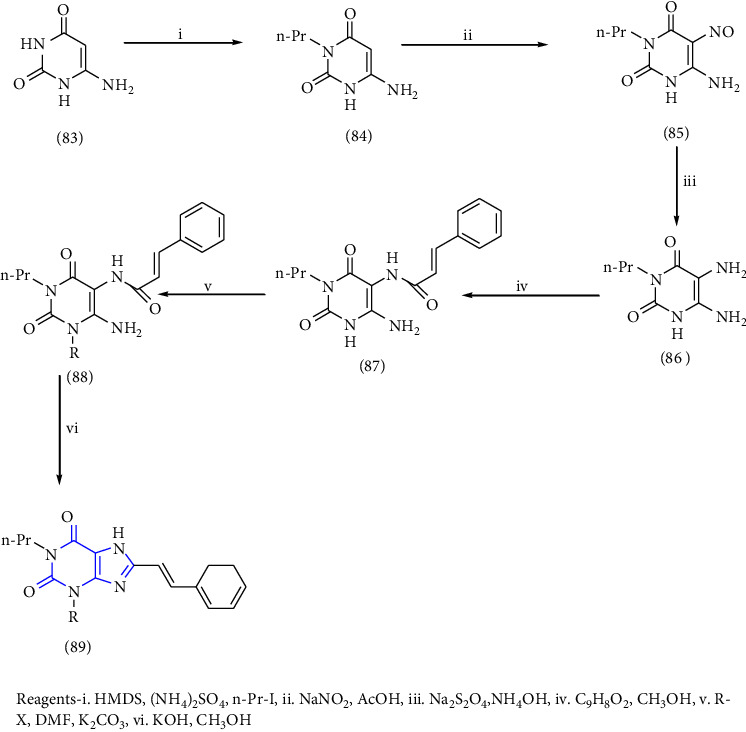
1,3-disubstituted 8-styrylxanthines.

The synthesis of 1,3-disubstituted-8-styrylxanthines under chemo and regioselective conditions was reported. The synthesis was achieved by using 6-aminouracil **(83)** as the starting material. The 6-aminouracil was alkylated at 3-position to form 6-amino-3-propyluracil **(84)**, which undergo nitrosation **(85)** and reduction to yield 5,6-diamino-3-propyl uracil **(86)**. The obtained compound was condensed with EDCI and cinnamic acid to form 6-amino-3-propyl-5-styrylcarboxamide **(87,88)** and finally, cyclisation was achieved by using alkali to form the resultant xanthine derivative **(89)**. All the synthesized compounds have shown good activity for both A1 and A2A adenosine receptors [34] (Scheme 13).

### 3.2. Xanthine-Anneleated Synthesis

**Scheme 14:**
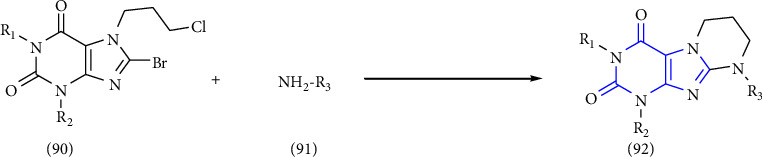
Synthesis of tetrahydropyrimido [2,1-f] purinediones using the convergent approach.

In xanthine-anneleated synthesis, the upgradation of one more ring starting from the bicyclic xanthine scaffold produces several biologically important compounds with modified physiochemical properties. Though, xanthine-anneleated synthesis is tedious but not widely used for the synthesis of xanthine derivatives. In this context, the synthesis of tetrahydropyrimido [2,1-f] purinediones by using a convergent approach was reported. The commercially available amines **(91)** were treated with 8-bromo-7-(3-chloropropyl)-1,3-dialkylxanthine **(90)** in presence of a base in DMF (dimethyl formamide) to form the substituted xanthine derivatives **(92)** [35] (Scheme 14).

**Scheme 15:**
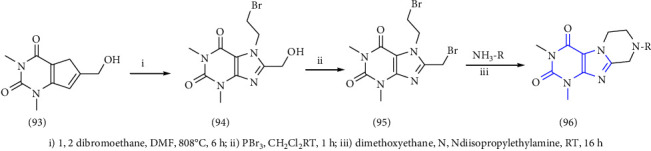
Synthetic pathway towards 8-substituted-purine-2,4(1H,3H)-diones.

Another contribution to this study is based on the synthesis of 8-Benzyl-substituted tetrahydropyrazino [2,1-f] purinediones was reported. The 1,3-dimethyl-8-hydroxymethylxanthine **(93)** was used as the starting material in this study. Primarily, position-7 of xanthine was alkylated **(94)** with 1,2 dibromoethane. The hydroxy group present at the 8-position was then converted into the corresponding bromide to form the resulting purinedione derivative **(95)** which was further treated with different substituted benzylamines to afford the tetrahydropyrazino derivative **(96)** [36] (Scheme 15).

**Scheme 16:**
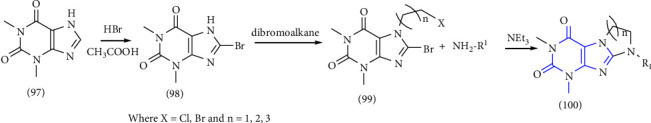
Synthesis of tricyclic xanthine derivatives.

Another series based on xanthine anneleated synthesis was reported. The N-9 benzyl substituted purinediones were synthesized from theophylline **(97)**, which was oxidatively brominated to give the compound **(98)**. Subsequently, 8-bromotheophylline has been alkylated at the N-7 position to obtain an 8-bromo-7-alkyltheophylline derivative **(99)**. Finally, condensation with appropriate amine leads to tricyclic xanthines **(100)** [37] (Scheme 16).

**Scheme 17:**
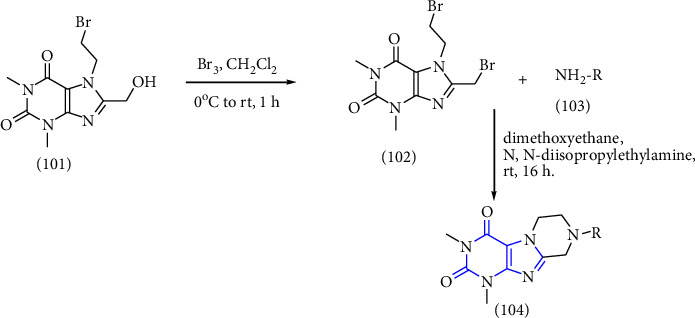
Synthesis of N8-substituted 1,3-dimethyltetrahydropyrazino [2,1-f] purinediones.

Moreover, 8-Substituted 1,3-dimethyltetrahydropyrazino [2,1-f] purinedione derivatives were reported as multitargeted drugs. Initially, the compound **(101)** was converted to the corresponding bromide to get compound **(102)**. Furthermore, the compound was treated with appropriate amine **(103)** to afford tetrahydropyrazino derivatives **(104)** [38] (Scheme 17).

### 3.3. One-Pot Method

**Scheme 18:**
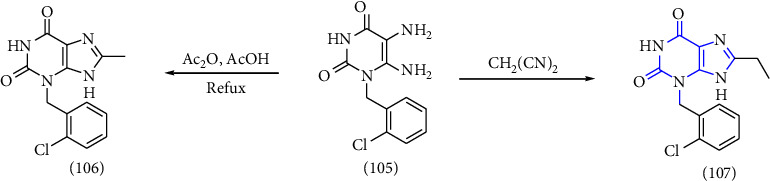
One-pot synthesis of xanthines.

According to the emerging importance of xanthine and its derivatives. The development of a novel method for access to xanthine scaffold in less time, without using toxic chemicals, and improvement in yield is still in great demand. In this study, numerous functionalized xanthines were synthesized with better yield, without the use of toxic reagents, and in lower time. Based on the aforementioned information, a one-pot synthesis of xanthine was reported. The 5,6-diaminouracil **(105)** was chosen as the substrate. The substrate was refluxed with acetic anhydride in acetic acid to obtain compound **(106)** and heating of substrate with malononitrile gave compound **(107)** in good yield [39] (Scheme 18).

**Scheme 19:**
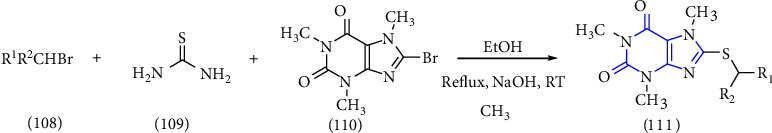
One-pot three-component reaction-alkyl bromide, thiourea, and 8-bromocaffeine.

In addition, the synthesis of some 8-alkylmercaptocaffeine derivatives has been reported via a one-pot three-component reaction. The treatment of thiourea **(109)** with alkylbromide **(108)** and 8-bromocaffeine **(110)** yielded 8-alkylmercaptocaffeine derivative **(111)** in excellent yield [40] (Scheme 19).

**Scheme 20:**
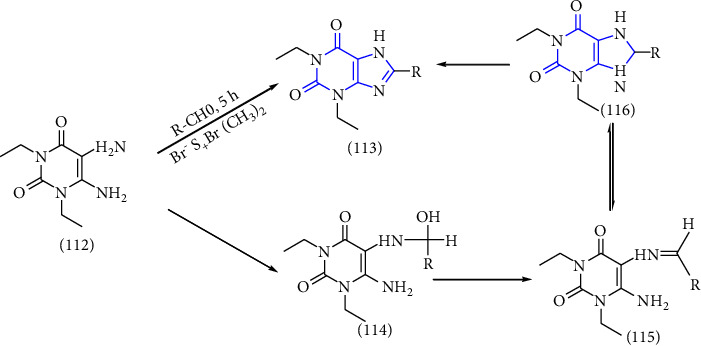
One-pot method for the synthesis of xanthine core via BDMS accelerated condensation.

Another one-pot synthesis of 8-xanthine derivatives was reported. The synthesis was done by treatment of 5,6-diaminouracil **(112)** with a simple aldehyde to form a xanthine derivative **(113,114)** through an imine intermediate **(115)**. Furthermore, cyclisation of the intermediate yield xanthine derivative **(116)**. All the synthesized compounds were observed for A2A adenosine receptor antagonist [41] (Scheme 20).

### 3.4. Miscellaneous Synthesis

**Scheme 21:**
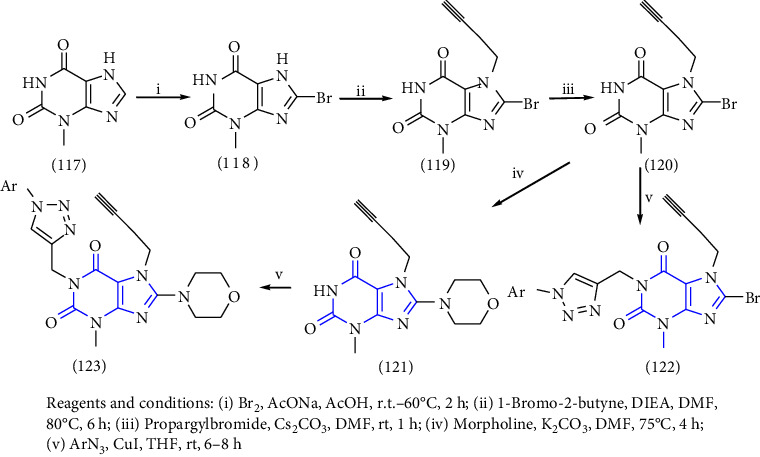
Synthesis of 1,2,3-triazole-based xanthines.

Xanthines can also be used as a starting material for the large-scale manufacture of xanthine derivatives. Mainly the alteration at 1-, 3-, 7- and 8- in the xanthine scaffold results in derivatives with numerous pharmacological activities. To discover numerous roles of xanthines, the examination of various methods used for the synthesis became an emerging topic of research. In this context, a novel series of 1,2,3-triazole-based xanthines were designed and synthesized. The compound **(117)** was treated with bromine to form an intermediate **(118)**. The intermediate was then allowed to react with 1-bromo-2-butyne gave compound **(119)**, subsequent interaction of this compound with propargyl bromide gave another purine intermediate **(120)** which on treatment with morpholine gave morpholinodione intermediate **(121)**. Lastly, the addition of the 1,3-dipolar cycle of terminal alkyne gave the corresponding 1,2,3-triazoles **(122,123)** in good yield [42] (Scheme 21).

**Scheme 22:**
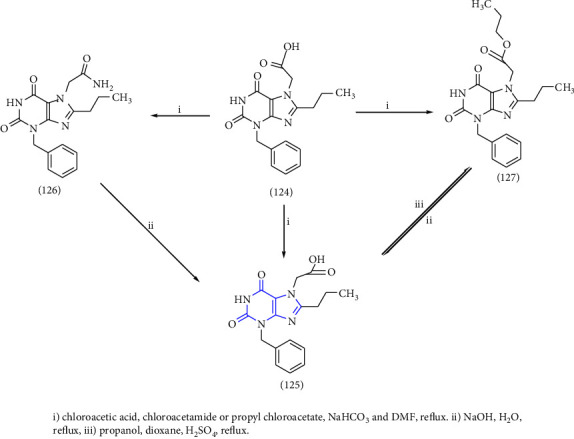
Synthesis of 3-benzyl-8-propylxanthinyl-7-acetic acid, its ester, and amide.

3-benzyl-8-propylxanthinyl-7-acetic acid and its derivatives were designed, synthesized, and reported. The compound **(124)** was taken as a lead compound. The lead compound was treated with chloroacetic acid, chloroacetamide, or propyl chloroacetate to form the target xanthine derivatives **(125-127)** [43] (Scheme 22).

**Scheme 23:**
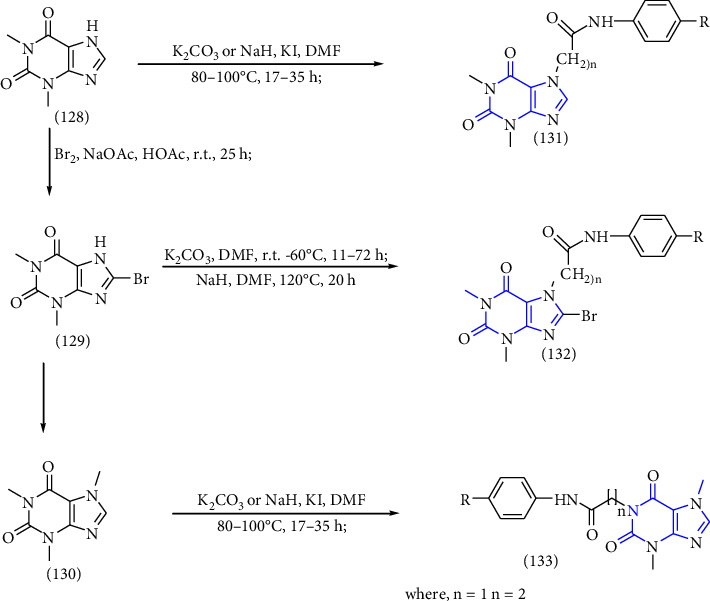
Schematic synthesis of xanthine derivatives.

Theophylline **(128)**, 8-bromotheophylline **(129),** and theobromine **(130)** were reacted with various 2/3-chloro-N-phenylacetamides or their propanamide analogs to obtain the resultant xanthine derivatives **(131-133)**. The synthesized compounds were biologically assessed for in vitro bronchodilator activity [44] (Scheme 23).

## 4. Reported Potent Compounds of Xanthines and Their Targets

Natural and synthetic compounds consisting of xanthine scaffold showed a variety of pharmacological activities. A large number of biologically active compounds were obtained by incorporating different substituents at different places in this ring [22]. A number of reported potent compounds of xanthines along with the therapeutic disease target are discussed (Table 2).

## 5. Conclusion

Our prime aim in writing this review paper is that the content presented in this review paper will be beneficial to the field and will provide great help to those researchers working on this scaffold. This review article provides a summary overview of the synthesis of xanthine structures. The development of novel, selective and efficient methods for the formation of xanthine ring starting from commercially available substrates is a pivotal target in the current organic synthesis. In this regard, the synthesis of xanthine derivatives by traube's method is the most used approach. However, other approaches were also found attractive to researchers. The various synthetic procedures exemplified in this review paper may serve as a support system for the designing of new molecules with xanthine scaffold. We hope that the data compiled in this review paper could help the medicinal chemist in designing new active compounds from the modification of the already existing compounds in the search for novel drug leads.

## Figures and Tables

**Table 1:** Patent filed for xanthine derivatives in last twenty years (2002–2020).

S. No.	Patent application number	Year of filing	Applicant name	Patent office	Compound	Category
1	AU 200521 958-B2 [6]	2005	Boehringer Ingelheim International GmbH	Australian	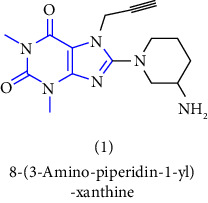	DPP-IV inhibitor

2	CA 02873524 [7]	2007	Boehringer Ingelheim International GmbH	Canadian	1-[(4-methyl-quinazolin-2-yl)methyl]-3-7-(2-butyn-1-yl)-8-(3-(R)-aminopiperidin-1-yl) xanthine	DPP-IV inhibitor

3	EP 2058311A2 [8]	2003	Boehringer Ingelheim International GmbH and Co.	European patent office	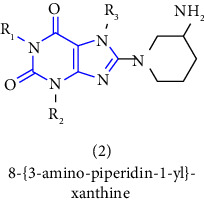	DPP-IV inhibitor

4	DE 6020050 00 986T2 [9]	2005	Loreal Paris	Deutsches patent under Markenamt	—	Obesity

5	EP 1368349 B1 [10]	2002	Boehringer Ingelheim International GmbH and Co.	Europian	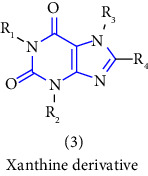	DPP-IV

6	EP 1515972 B1 [11]	2003	F.Hoffman-La Roche AG 4070 Basel (CH)	Europian	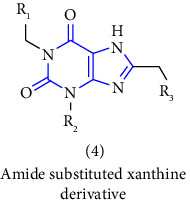	Type-II diabetes

7	EP 1599477 B1 [12]	2004	F.Hoffman-La Roche AG 4070 Basel (CH)	Europian	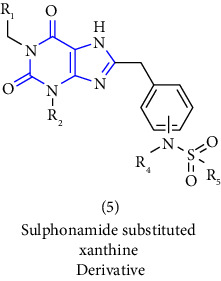	PEPCK inhibitors

8	EP 1689748 B1 [13]	2004	Boehringer Ingelheim International GmbH	Europian	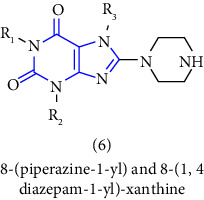	Diabetes

9	ES 2401128 T3 [14]	2006	GlaxoSmithKline LLC	Spain	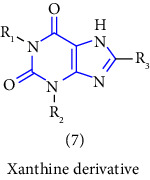	Agonist of HM74 A

10	US 7696212 B2 [15]	2010	Boehringer Ingelheim International GmbH and Co. KG	United States	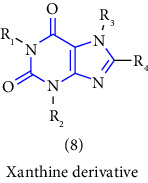	DPP-IV inhibitor

11	US 7838529 B2 [16]	2010	Boehringer Ingelheim International GmbH, Ingelheimam Rhein (DE)	United States	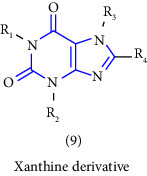	Type-2 diabetes mellitus, antiobesity.

12	US 7879864 B2 [17]	2011	Sanofi-Aventis Deutschland GmbH, Frankfurt am main (DE)	United States	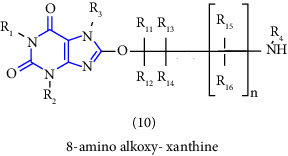	DPP-IV inhibitor

13	US 9221821 B2 [18]	2015	Forest Laboratories Holdings Limited, Hamilton (BM)	United States	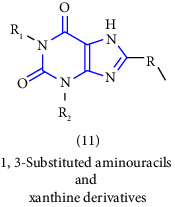	-

14	US 10202383 B2 [19]	2019	Boehringer Ingelheim International GmbH, Ingelheim am Rhein (DE)	United States	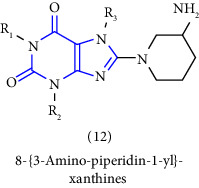	DPP-IV

15	US 10214530 B2 [20]	2019	Max-Delbruck-Centrum Fur Molekulare Medizin, Berlin (De), Forschungs-Verbund-Berlin-E. V., Berlin (De)	United States	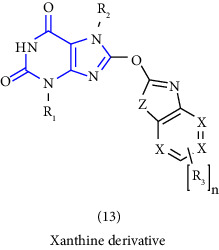	Serotonin based disease

16	US 201111 8464 [21]	2009	Ing-Jun Chen Linya District (TW)	United States	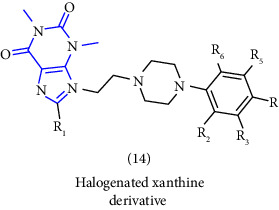	Anticancer

**Table 2:** Reported potent compounds of xanthines and their targets.

S. No.	Substituted xanthine derivative	Disease target	Potent compounds reported
1	1,3,8- and 1,3,7,8-substituted xanthines derivatives [23]	A2B and A1 adenosine receptors	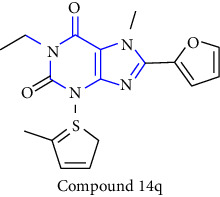

2	8(cyclopentyloxy) phenylxanthines derivatives [24]	A1 and A2 adenosine receptors	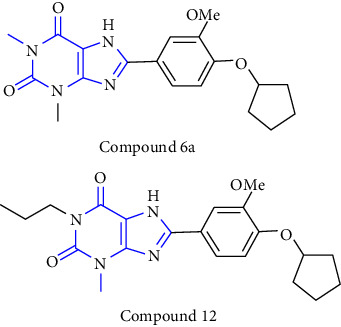

3	8-(phenoxymethyl) xanthine and 8-(3-phenylpropyl) xanthine derivatives [25]	A2A adenosine receptor antagonistic properties	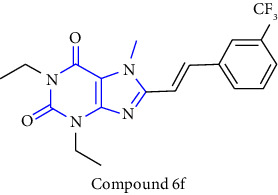

4	8-(p-substituted-phenyl/benzyl) xanthines derivatives [26]	A2A adenosine receptor	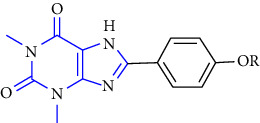

5	1,3-diethyl-7-methyl-8-(phenoxymethyl)-xanthine and 1,3,7-trimethyl-8-(phenoxymethyl)-xanthine derivatives [27]	A1 and A2A adenosine receptor antagonists	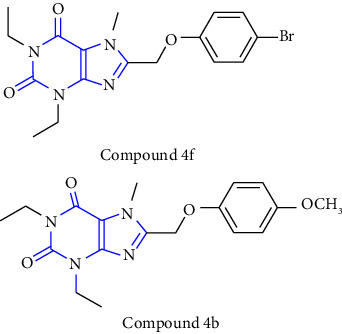

6	8-(1-prop-2-ynyl-1*H*-pyrazol-4-yl)-xanthine derivatives [28]	A2B adenosine receptor antagonists	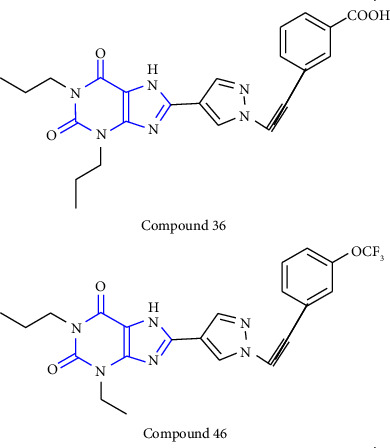

7	Tetrahydropyrazino-annelated theophylline derivatives [29]	Multi-targeted drugs with adenosine receptor (A1, A2A) and MAO-B antagonistic activity.	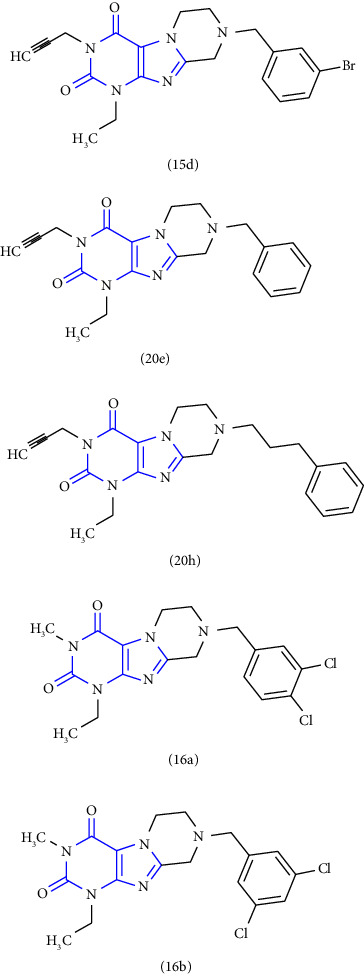

8	8-(2-nitroaryl) xanthines [30]	Human A2A adenosine receptor	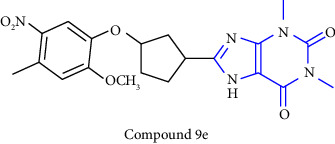

9	8-(3-phenylpropyl) xanthines 8-(2-phenylethyl) xanthines and 8-(phenoxymethyl) xanthines [31]	Adenosine A1 receptors	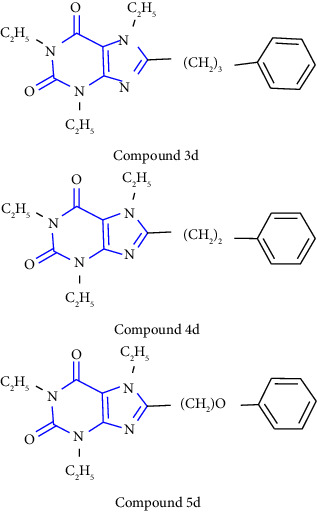

10	Carboxylate amides of 8-phenyl-1,3-dimethylxanthine [32]	Adenosine A2A receptors	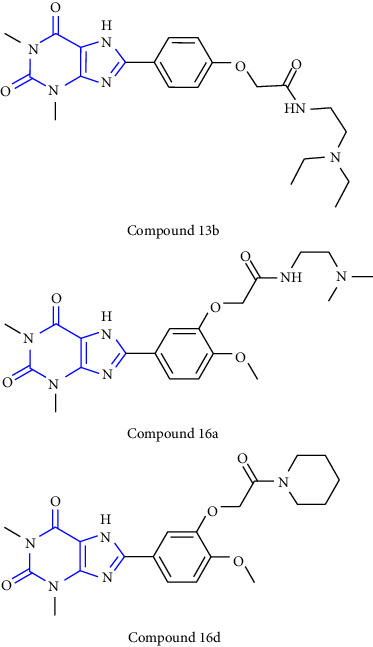

11	6-amino-5-carboxamidouracils [33]	—	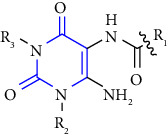

12	1,3-substituted 8-styrylxanthines [34]	A1 and A2A adenosine receptors antagonist	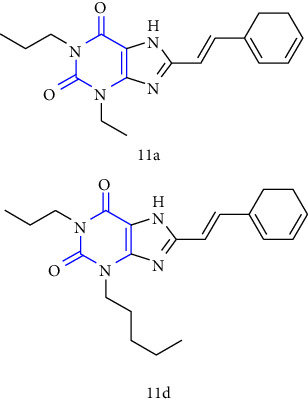

13	Tetrahydropyrimido[2,1-*f*] purinediones derivatives [35]	A2B adenosine receptor antagonists	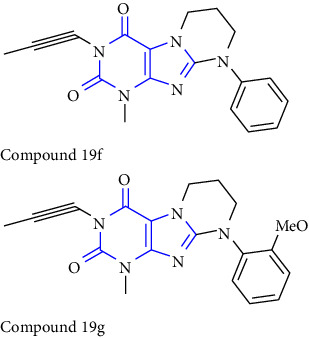

14	8-benzyltetrahydropyrazino [2,1-f] purinediones derivatives [36]	Dual-target-directed A1/A2A adenosine receptor antagonists	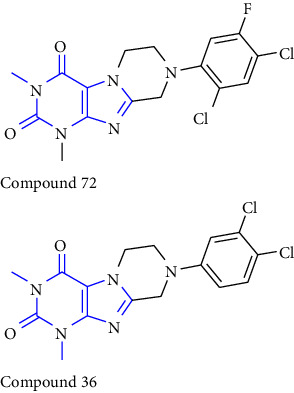

15	1,3-dialkyl-substituted tetrahydropyrimido [1,2-f]purine-2,4-diones [37]	Human A2A adenosine receptor antagonists	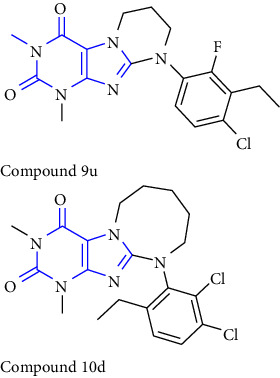

16	8-benzyl-substituted tetrahydropyrazino [2,1-f] purinediones [38]	Dual A1/A2A adenosine receptor antagonists	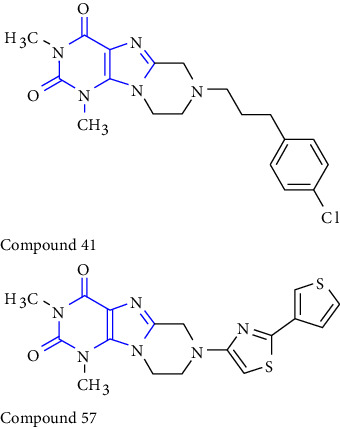

17	Xanthine derivatives [39]	Antimicrobial and antioxidant activities	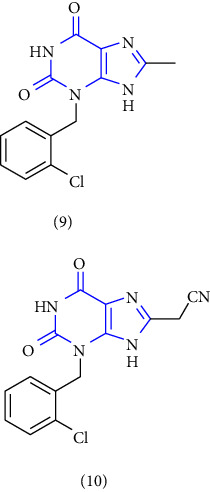

18	8-alkylmercaptocaffeine derivatives [40]	—	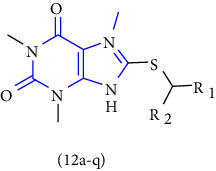

19	8-substituted xanthine derivatives [41]	A2A adenosine receptor antagonists	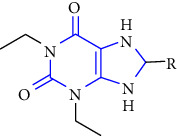

20	1,2,3-triazole-based xanthine derivatives [42]	Dipeptidyl peptidase-4 inhibitors	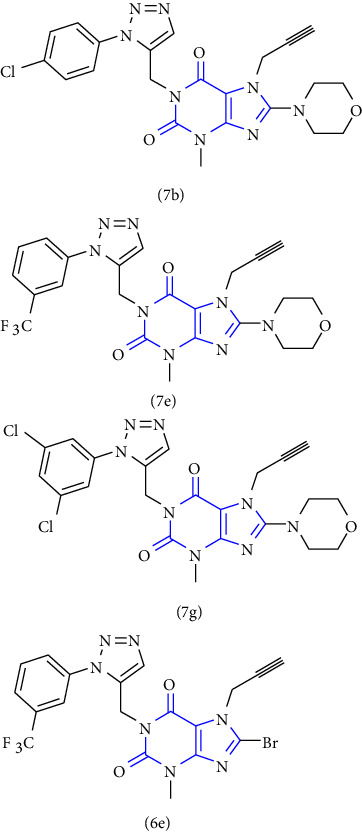

21	3-benzyl-8-propylxanthinyl-7-acetic acid [43]	—	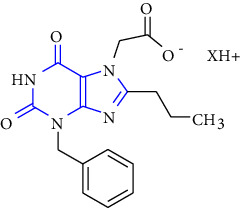

22	1,3,8-substituted tetrahydropyrazino [2,1-f] purinediones [44]	A1 and A2A adenosine receptors and MAO-B	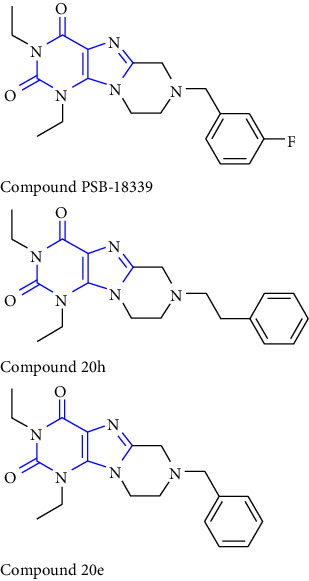

## Abbreviations

DMF: Dimethylformamide
SAR: Structural activity relationship
*A*_1_, *A*_2*A*_, *A*_2*B*_, *A*_3_: Adenosine receptor subtypes
EtOH: Ethanol
H_2_O: Water
HCl: Hydrochloric acid
NaNO_2_: Sodium nitrite
AcOH: Acetic acid
HCl: Hydrochloric acid
HCOOH: Formic acid
HCOONa: Sodium formate
NaOH: Sodium hydroxide
HC(OEt)_3_: Triethylorthoformate
CH_3_I: Methyliodide
K_2_CO_3_: Potassium carbonate
MeOH: Methanol
COOH: Carboxylic acid
SOCl_2_: Thionyl chloride
RT: Room temperature
DIPEA: Diisopropylethylamine
NaH: Sodium hydride
KI: Potassium iodide
C_2_H_5_I: Ethyl iodide
CH_3_COOH: Acetic acid
HBr: Hydrogen bromide
NaClO_3_: Sodium chlorate
TEBA: Benzyltriethylammonium chloride
NEt_3_: Triethylamine
*µ*M: Micromolar
CH_2_Cl_2_: Dichloromethane
Ac_2_O: Acetic anhydride
CH_2_(CN)_2_: Malononitrile
AcONa: Acetic anhydride-sodium acetate
DIEA: Diisopropylethylamine
Cs_2_CO_3_: Cesium carbonate
CuI: Copper iodide
THF: Tetrahydrofuran
NaHCO_3_: Sodium hydrogen carbonate
H_2_SO_4_: Sulfuric acid
EDC HCl: 1-(3-Dimethylaminopropyl)-3-ethylcarbodiimide hydrochloride
EDCI: 1-Ethyl-3-(3-dimethylaminopropyl) carbodiimide.
